# Notes of the Week

**Published:** 1921-04-16

**Authors:** 


### NOTES OF THE WEEK.

### THE LEGALITY OF MUNICIPAL HOSPITALS.

Sir Alfred Mond had not long been in his new office before he heard about the Bradford Municipal Hospital Scheme, and he, as was his predecessor in the Health Ministry, was unable to speak off-hand as to the legality of the step, when challenged by a question in the Commons. The Westminster Board of Guardians has been asked by H.R.H. Princess Christian, as President of the Workhouse Nursing Association, to allocate wards in the infirmary to the general public impoverished by the war, and has, in expressing sympathy, come to the conclusion that it has no legal power to take up the scheme. This answer is probably given in view of a recent Ministry of Health circular to Boards of Guardians on the subject.

### THE APPROVED SOCIETIES.

In spite of some annoyance felt at the attitude of certain hospitals in proposing not to treat members of approved societies except upon conditions, the situation seems to be more favourable to a satisfactory settlement. No one now, even from the approved societies, denies that the hospitals have a claim upon them; it is only the extent of the claim and the way in which it can best be met that is in question. Opinion seems to favour some *pro rata* grant or payment, and this is probably a good solution, if the adjustment of the grant in each case does not lead to too much clerical work, too many forms, and to too much correspondence. We should welcome suggestions on these points, since the most should be made of the present favourable opportunity. The principle conceded, it is now the time for a frank discussion of the details.

### THE NEW ANÆSTHETIC.

There is considerable satisfaction to be derived from the report that Dr. R. Mackenzie Wallis of St. Bartholomew's Hospital has, by elimination of certain impurities, apparently produced a relatively safe anæsthetic of the ether class. Possibly the so-called "safety" of the substance will not in practice be of so much value or be so greatly appreciated by anæsthetists as the other benefits which its use is claimed to confer. Absence of bronchial irritation, elimination of post-anæsthetic vomiting, and its availability for patients who cannot safely and justifiably be treated under existing forms of anæsthesia, would alone give the new product outstanding claims to attention. Full technical details of the inventor's work will be received with the greatest interest; but experience shows that it is best not to pitch expectation too high in these matters.

### MEDICINE IN BRUSSELS.

In great part due to a munificent contribution by the Rockefeller Foundation (New York), funds up to 100 million francs are to be devoted to new buildings and endowments for the Medical School of Brussels University. The laboratory and classrooms of the Medical School will be completely rebuilt and redeveloped on a new site. The Municipal Hospital of St. Pierre will also be completely reorganised to serve as the teaching hospital of the University and will contain about 350 beds. Brussels had, before the war, established herself as a centre of European medical progress, and it is more than satisfactory that all facilities for rejuvenation in this respect will now be at hand.

### RESEARCH IN TROPICAL DISEASE.

This important sphere of national medicine stands to gain greatly by the action of the London and Liverpool Schools of Tropical Medicine in giving their support to plans for broadening the Gorgas Memorial Institute at Panama into a research and teaching foundation of international scope. In America, one is glad to see that the Harvard and Johns Hopkins Universities and the Rockefeller Foundation have all expressed their willingness to co-operate in the scheme.

### FILTERING OF TUBERCULAR CASES.

A correspondent to the *Daily Telegraph* pleads for more hospital accommodation where cases suspected of being tubercular may be received for preliminary investigation and classification. He therefore thinks, if the recent amalgamation of the Great Northern Central Hospital and the Royal Hospital for Diseases of the Chest means that the building of the latter institution will eventually be pulled down, that before any steps are taken it should be considered whether the hospital cannot, through an arrangement with the London County Council, be retained for the essential work of the filtering of real cases of tuberculosis, who alone require to be isolated and treated along the necessarily expensive sanatorium lines.

### WARRANTIES UNDER THE FOOD AND DRUGS ACTS.

Another effort is to be made to induce the Government to repeal the warranty clauses in the Sale of Food and Drugs Acts. These have become a source of danger to the public, especially in the case of milk, because the guarantor is not in a position to control the article guaranteed in all its stages of transport. The efforts of the municipal authorities to obtain a pure milk supply have consequently been thwarted, but a combined effort is now being made by them to obtain an alteration in the law. Battersea Borough Council called a conference to discuss this question, to which twenty Metropolitan Councils sent representatives. They were unanimous in condemning the present position, and passed a resolution requesting the Government to take all necessary steps without delay to repeal the warranty clauses in the Sale of Food and Drugs Acts, whilst a deputation was appointed to wait on the Minister of Health urging him to take immediate action.

### A GREAT FLOATING HOSPITAL.

Under the appropriate name of *Relief* the newest United States hospital ship has joined the Atlantic Fleet. It is claimed to be the last word in floating hospitals, and the first vessel in the navies of the world built from the keel up as a hospital ship. It has accommodation for 500 patients, is equipped with every recent device for safety and care of sick and wounded, including six operating-rooms, and has all the facilities of a great modern hospital on land. The American Navy has three other hospital ships which have been converted from merchant ships, and this service fulfils all hospital requirements of the different fleets, and at the same time supplies a medicine depôt for the use of the individual ships. The *Relief* carries in her hold a complete field hospital, with surgical instruments, dressings, drugs, etc., and can at any time land a party on shore with a field hospital ready for service. This, of course, would answer other purposes besides those of war. In such an eventuality as the Baltimore fire or the San Francisco earthquake the field hospital would be of the greatest service.

### NEW SECRETARY OF WESTMINSTER HOSPITAL.

A successor to Mr. Sidney M. Quennell at Westminster Hospital has now been appointed in the person of Captain C. M. Power, of St. Bartholomew's Hospital. It will be remembered that Capt. Power was in 1919 chosen to succeed the late Mr. Arthur Watkins as steward of St. Bartholomew's Hospital, and thus has had about two years' experience of voluntary hospitals.

### THE CULTIVATION OF SUNDAY SCHOOLS.

The strenuous efforts which are being made in Manchester to raise money for the hospitals there, and the preparations for Hospital Sunday, have led a worker in the Sunday schools to suggest that £10,000 could easily be raised from these schools alone. He has informed the *Manchester Dispatch* that his own school raised £25 in two weeks through "the simple efforts of about one hundred boys' and girls' enthusiastic collecting of pennies from parents and friends." Each quarter a "silver star for service" is awarded for the most successful effort, and "H. R. A." suggests that 400 similar efforts could be made, and, therefore, £10,000 be raised in a week or two. It still remains to be proved that 400 such collections could be carried through without difficulty.

### TREATMENT OF LIMBLESS IN WALES.

Mr. W. Percy Miles, Chairman of the Executive Committee of the Prince of Wales's Hospital (which has dealt with all limbless sailors and soldiers resident in Wales and Monmouthshire since it was opened on May 7, 1917), has received from the War Office the following letter, viz.:—"Now that the Prince of Wales's Hospital, Cardiff, has ceased to be used for military patients, the Army Council would take this opportunity of expressing their appreciation of the splendid work in the treatment of members of His Majesty's Forces which the hospital carried out during the period 1914-1920, and would be glad if this expression of their thanks, rendered on behalf of the nation, could be conveyed to the staff of the hospital for the excellent services which they so ungrudgingly rendered." The hospital is a remarkable institution in many ways, and has provided a centre of ideas for the treatment of this class of patients. The Secretary, Mr. Gilbert D. Shepherd, has worked hard, and the staff and himself will appreciate this recognition.

### SUPERVISION OF BLIND CHARITIES.

The Charity Commissioners for England and Wales, in making their sixty-eighth report, referred to the winding up of war charities, in respect of which a number of schemes have been presented, and finally approved, if they provide that the surplus funds involved be devoted to an object similar to the original object of the Charity. Under the Blind Persons Act passed last year the War Charity Act was, subject to certain modifications, applied to each charity, and the subscribing public are protected by the regulations in regard to the registration and public supervision contained in the Act. The total number of War Charities registered since the Act came into operation in 1919 is 11,907.

### HOSPITAL LIFE IN CORK.

It is remarkable that the Victoria Hospital, Cork, has gone through the past year with financial improvement, partly thanks to the popularity of the private rooms for male patients, which are proving of great value to the district, and a modest source of revenue as well. Even here, however, the dire conditions of the country have interfered: during a large part of last year the South of Ireland was paralysed by a railway crisis, which largely prevented outlying districts from sending patients into the city. The Council looks upon the future with anxiety because, "while the rest of the kingdom is gradually making good the ravages of war, the greater part of our land has unhappily lapsed into a state of chaos," and "where the business of a country declines charitable institutions cannot continue to flourish." In these circumstances the hospital deserves well for what it has done.

### RECORD SATURDAY FUND COLLECTION.

At the annual meeting of the Metropolitan Hospital Saturday Fund held at the Mansion House last Saturday a most satisfactory account was given of the past year's work. For the first time the Earl of Malmesbury, Chairman of the Council, took the chair, and he said that, in spite of the industrial unrest, 1920 had been a record year for the Fund; the income amounted to no less than £106,455, or £31,854 in excess of the previous record collection in 1919. This splendid result was undoubtedly in a large measure due to the devoted services of the local committees, without which the Fund would certainly have suffered. An annual collection of at least £150,000 is the present aim, and it is quite likely to be achieved, as the receipts for the first quarter of 1921 increased by £3,600 as compared with last year. The meeting was altogether an interesting one, during which Sir Arthur Stanley made a presentation of the Order of St. John of Jerusalem to Mr. Joseph Ash, and Sir William Collins gave an address on "The Voluntary Hospitals." Everybody is agreed that the hospitals must appeal to the multitude as well as the moneyed few, and the Hospital Saturday Fund is helping them to do it.

